# Late-Onset Pancreatic Metastasis From Renal Cell Carcinoma Mimicking a Neuroendocrine Tumor: A Diagnostic Challenge and Literature Review

**DOI:** 10.7759/cureus.90158

**Published:** 2025-08-15

**Authors:** Manoj Jain, Anurag Singh, Nirbhay Kumar, Samir Mohindra

**Affiliations:** 1 Pathology, Sanjay Gandhi Postgraduate Institute of Medical Sciences, Lucknow, IND; 2 Gastroenterology, Sanjay Gandhi Postgraduate Institute of Medical Sciences, Lucknow, IND

**Keywords:** metastasis, nephrectomy, pancreatic body, pancreatic malignancy, renal cell carcinoma

## Abstract

Pancreatic metastasis from renal cell carcinoma (RCC) is a rare and often delayed event, frequently resembling primary pancreatic neoplasms such as neuroendocrine tumors, which poses substantial diagnostic challenges. This report describes a case involving a 74-year-old male patient with pancreatic metastasis originating from the left kidney. Seven years earlier, the patient had undergone a left nephrectomy for RCC. Follow-up evaluations after seven years revealed a mass in the pancreatic body, with initial radiological assessments suggesting a provisional diagnosis of a neuroendocrine tumor. Endoscopic ultrasound-guided fine-needle biopsy of the pancreatic lesion, combined with a panel of immunohistochemistry markers, confirmed that the mass was metastatic RCC. A surgical procedure is planned for the patient. This case report presents an uncommon instance of delayed pancreatic metastasis from RCC and reviews the relevant literature on its clinical presentation, diagnostic approaches, and treatment options.

## Introduction

Recent advances in diagnostic methods indicate that nearly one in four solid pancreatic lesions is not ductal adenocarcinoma, challenging the previous belief that all pancreatic masses were carcinomas. Current differential diagnoses include inflammatory masses, lymphomas, neuroendocrine tumors, and metastases [1]. The pancreas is an uncommon site for metastasis compared with other retroperitoneal abdominal organs. Pancreatic metastases are rarely observed in clinical practice, representing approximately 2% of all pancreatic malignancies. They are most often caused by renal cell carcinoma (RCC), melanoma, and malignancies originating from the breast, ovaries, and colon [2,3]. Metastatic RCC in the pancreas is associated with a notably favorable outcome, in contrast to the generally poor prognosis linked to metastatic disease [4]. This report describes a case of a 74-year-old male patient with delayed RCC metastasis to the pancreas, which primarily mimicked a neuroendocrine tumor on radiology, and includes a review of the literature, emphasizing its clinicopathological features and current management protocols.

## Case presentation

**Figure 1:**
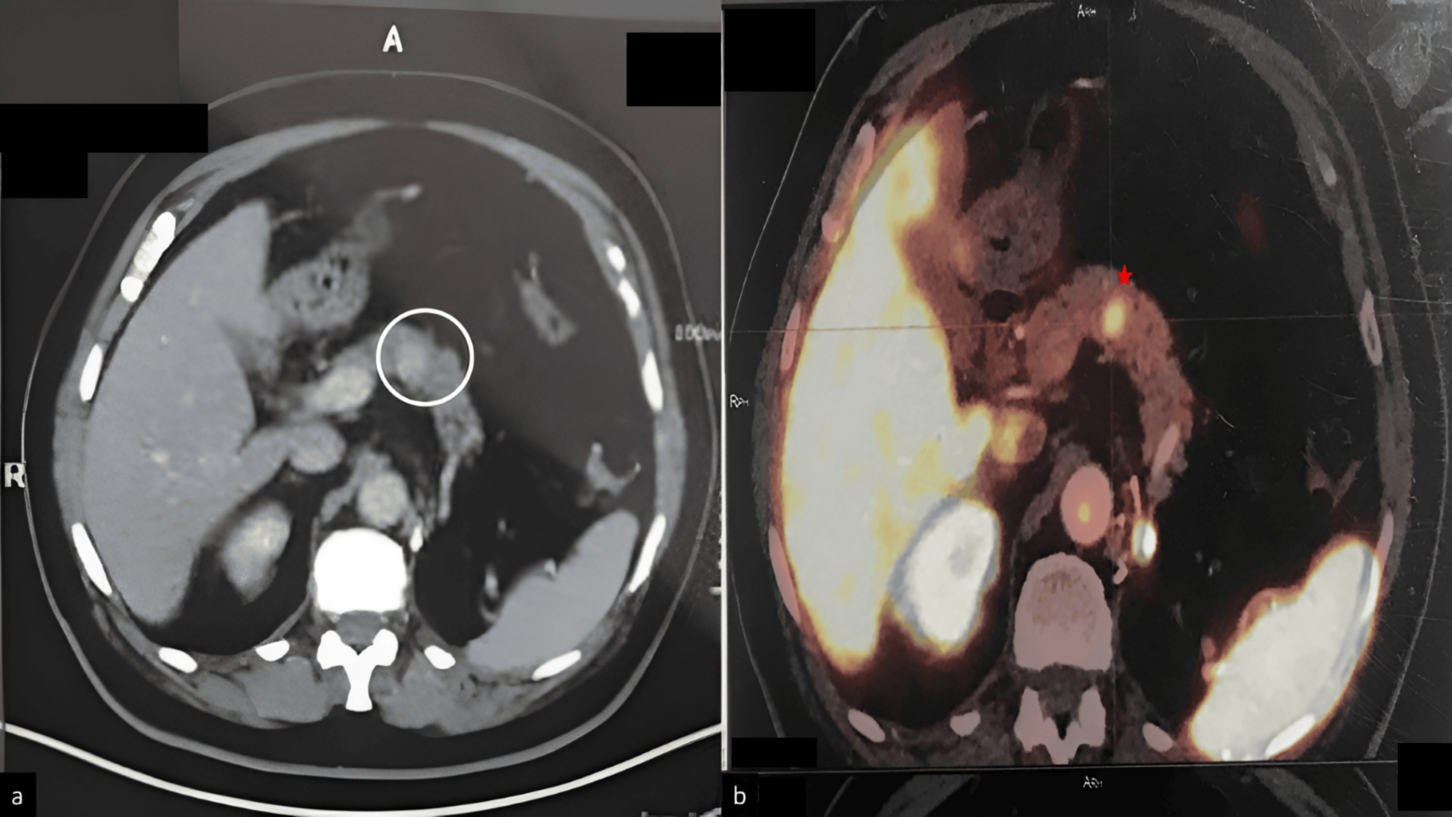
(a) Contrast-enhanced CT of the abdomen showing a hyperenhancing area measuring 18 × 15 mm in the body of the pancreas (encircled). (b) PET scan displaying focal uptake in the proximal body of the pancreas* and no evidence of residual disease at the postoperative site (left iliac fossa).

A 74-year-old male patient with a history of clear cell RCC presented with unexplained weight loss and vague abdominal discomfort. He had a history of hypothyroidism and was on a daily oral dosage of 25 mg thyroxine. Seven years earlier, he had undergone a left nephrectomy for a renal mass, which on histopathology was reported as clear cell type RCC (Fuhrman grade II). He did not receive any adjuvant therapy. Physical examination revealed a postoperative abdominal scar and tenderness in the upper abdomen; however, other systemic evaluations were normal. Contrast-enhanced CT of the abdomen revealed an isolated hyperenhancing mass measuring 18 × 15 mm in the pancreatic body, suggesting primarily a neuroendocrine tumor of the pancreas; however, the possibility of metastatic RCC, given the patient’s history, was kept as a differential diagnosis (Figure 1a). A whole-body PET scan was performed using a Umi 550 digital PET/CT (United Imaging Healthcare, Shanghai, China) after injecting F-18 fluorodeoxyglucose, which showed a focal area of activity in the pancreas but no evidence of residual or recurrent disease at the surgical site in the left iliac fossa. No other hypermetabolic lesion was identified anywhere in the body (Figure 1b).

**Figure 2:**
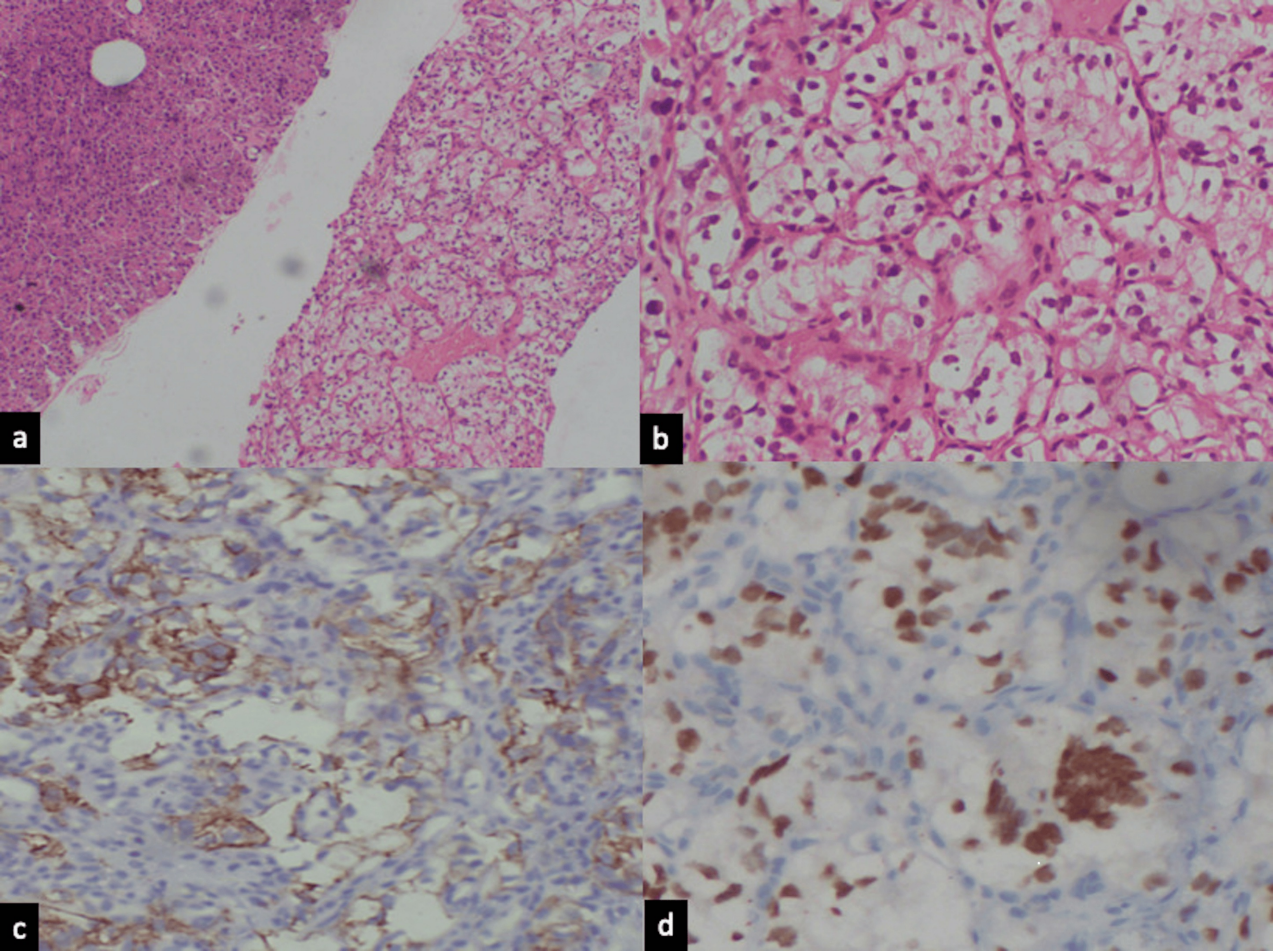
(a) Ultrasound-guided FNB of the pancreas showing fragments of pancreatic tissue and tumor cells arranged in sheets and nests separated by thin fibrovascular septae (H&E stain, 10×). (b) Metastatic tumor cells exhibiting moderate pleomorphism, with round to oval nuclei, dispersed chromatin, inconspicuous nucleoli, and abundant vacuolated clear cytoplasm (H&E stain, 400×). (c) Tumor cells showing positive expression of CD10 (400×). (d) Tumor cells showing positive expression of PAX8 (400×). FNB, fine-needle biopsy

An endoscopic ultrasound (EUS)-guided fine-needle biopsy (FNB) was performed, which showed pancreatic tissue fragments infiltrated by tumor cells organized in sheets and small nests separated by thin fibrovascular septae. The tumor cells exhibited moderate pleomorphism, with round to oval nuclei, dispersed chromatin, inconspicuous nucleoli, and moderate to abundant vacuolated clear cytoplasm (Figure 2a, 2b). A provisional diagnosis of metastatic clear cell RCC was made based on histomorphology and the patient’s previous history. To confirm the diagnosis, immunohistochemistry was performed, which revealed positive expression of CD10, PAX8, and vimentin, with no expression of synaptophysin, chromogranin, napsin, CK7, or CK20 (Figure 2c, 2d).

## Discussion

RCC can metastasize to multiple organs, including the pancreas [4,5]. Metastasis of RCC to the pancreas is associated with a favorable prognosis, suggesting a slower growth rate of the metastatic tumor compared with its spread to other organs [6]. This evidence has led authors to conclude that pancreatic metastases in RCC indicate a comparatively better clinical outcome. Metastases from RCC often respond well to treatment and are associated with longer survival, even though the presence of a mass in the pancreas usually signals worse outcomes in pancreatic ductal adenocarcinoma and neuroendocrine neoplasms. This is in stark contrast to the aggressive characteristics and chemoresistance of pancreatic cancer [7,8].

Metastatic spread to the pancreas from RCC primarily occurs via the bloodstream. Metastatic lesions may present as isolated or multifocal. The development of isolated pancreatic metastases in RCC is thought to be governed by a specific “seed-and-soil” mechanism, involving the selective embolization of cells from the primary tumor to the pancreas. This mechanism results in a uniform distribution of metastatic lesions throughout the pancreas, regardless of their RCC origin, leading to comparable outcomes for both simultaneous and sequential occurrences [9,10]. Metastatic spread to the pancreas from RCC can occur either concurrently or at a later time. Patients with a history of RCC require prolonged surveillance due to the potential for late metastasis [10].

Pancreatic metastases typically develop silently, exhibiting slow growth and less aggressive tumor biology. Metachronous metastasis to the pancreas from RCC may occur as long as thirty years after the diagnosis of the initial tumor [11,12]. In our case, pancreatic metastases appeared seven years post-nephrectomy. Initially, patients may present with symptoms such as gastrointestinal bleeding, pancreatitis, or jaundice. However, the detection of a pancreatic mass during follow-up or the onset of symptoms such as weight loss often leads to the diagnosis [13]. In this case, the patient first reported unexplained weight loss and general abdominal discomfort.

Abdominal imaging techniques, including CT and MRI, facilitate the detection of pancreatic malignancy. EUS improves lesion characterization and enables tissue sampling of a suspected pancreatic mass via FNB. Advanced imaging modalities, such as contrast-enhanced EUS and elastography, further improve the differentiation of solid pancreatic lesions. Metastases from RCC in the pancreas typically show hyperenhancement, similar to neuroendocrine tumors, whereas metastases from other primary tumors, such as colorectal or breast cancers, may appear iso- or hypo-enhanced [14].

Surgery, radiation therapy, and chemotherapy are available treatment options for pancreatic metastases originating from clear cell RCC. Compared with other metastatic sites, pancreatic metastases from RCC generally have a better prognosis. Surgical intervention remains the main treatment for oligometastatic disease involving the pancreas [14,15]. Various surgical procedures, including pancreatoduodenectomy, central pancreatectomy, distal pancreatectomy, and total pancreatectomy, are performed based on the tumor location. Targeted therapy and immunotherapy may be useful when disease progression occurs after surgical excision of metastases. Pancreatic metastases have shown notable responsiveness to tyrosine kinase inhibitors, with documented cases of complete radiologic responses [15].

## Conclusions

The pancreas is a recognized site for metastasis from RCC, which may present as a solitary or multiple lesions. Metastatic lesions can develop years after the initial diagnosis of the primary tumor, necessitating lifelong monitoring for patients with RCC. High-resolution imaging and radiology-guided tissue biopsy, combined with immunohistochemistry, are essential for the definitive diagnosis of a pancreatic mass.
